# Supplementary material for: Modeling glioblastoma heterogeneity as a dynamic network of cell states
Source: Mol Syst Biol. 2021 Sep 16;17(9):e10105. doi: 10.15252/msb.202010105 (PMC8444284; doi:10.15252/msb.202010105)
Supplement: Supplementary file 6 — Source Data for Figure 5 [file MSB-17-e10105-s004.zip › Figure5A_sourcedata/GSEA_3017/nosigdb_stateF.GseaPreranked.1622795015644/FELDMAN_2020_G2-M.html]

Details for gene set FELDMAN\_2020\_G2-M[GSEA]

|  || Dataset | state33017 |
| Phenotype | NoPhenotypeAvailable |
| Upregulated in class | na\_neg |
| GeneSet | FELDMAN\_2020\_G2-M |
| Enrichment Score (ES) | -0.6689457 |
| Normalized Enrichment Score (NES) | -5.046376 |
| Nominal p-value | 0.0 |
| FDR q-value | 0.0 |
| FWER p-Value | 0.0 |
Table: GSEA Results Summary

  

Fig 1: Enrichment plot: FELDMAN\_2020\_G2-M      
 Profile of the Running ES Score & Positions of GeneSet Members on the Rank Ordered List

  

| PROBE | GENE SYMBOL | GENE\_TITLE | RANK IN GENE LIST | RANK METRIC SCORE | RUNNING ES | CORE ENRICHMENT || 1 | CDKN1A |  |  | 4 | 2.089 | 0.0110 | No |
| 2 | CLU |  |  | 5 | 1.852 | 0.0225 | No |
| 3 | CST3 |  |  | 15 | 1.273 | 0.0260 | No |
| 4 | FTH1 |  |  | 19 | 1.203 | 0.0320 | No |
| 5 | S100B |  |  | 20 | 1.167 | 0.0393 | No |
| 6 | FTL |  |  | 32 | 1.036 | 0.0403 | No |
| 7 | IFITM3 |  |  | 38 | 0.987 | 0.0439 | No |
| 8 | SAT1 |  |  | 45 | 0.957 | 0.0469 | No |
| 9 | SPARC |  |  | 52 | 0.929 | 0.0498 | No |
| 10 | MALAT1 |  |  | 90 | 0.773 | 0.0363 | No |
| 11 | FILIP1L |  |  | 116 | 0.722 | 0.0285 | No |
| 12 | TP53I3 |  |  | 139 | 0.686 | 0.0219 | No |
| 13 | IGFBP7 |  |  | 164 | 0.643 | 0.0141 | No |
| 14 | ITM2B |  |  | 181 | 0.610 | 0.0100 | No |
| 15 | DDIT4 |  |  | 216 | 0.579 | -0.0031 | No |
| 16 | BTG1 |  |  | 222 | 0.574 | -0.0020 | No |
| 17 | TM7SF2 |  |  | 225 | 0.570 | 0.0005 | No |
| 18 | BEX4 |  |  | 242 | 0.559 | -0.0039 | No |
| 19 | MIAT |  |  | 263 | 0.542 | -0.0104 | No |
| 20 | FAM162A |  |  | 282 | 0.524 | -0.0160 | No |
| 21 | ATP6V0E1 |  |  | 303 | 0.510 | -0.0227 | No |
| 22 | PTPRZ1 |  |  | 310 | 0.505 | -0.0225 | No |
| 23 | PLD3 |  |  | 337 | 0.494 | -0.0322 | No |
| 24 | EIF4A2 |  |  | 380 | 0.478 | -0.0499 | No |
| 25 | EEF1A1 |  |  | 417 | 0.464 | -0.0648 | No |
| 26 | PSAP |  |  | 418 | 0.463 | -0.0619 | No |
| 27 | PRCP |  |  | 426 | 0.460 | -0.0625 | No |
| 28 | TTYH1 |  |  | 457 | 0.448 | -0.0745 | No |
| 29 | TCEAL8 |  |  | 471 | 0.444 | -0.0781 | No |
| 30 | BEX1 |  |  | 518 | 0.428 | -0.0981 | No |
| 31 | GPM6B |  |  | 553 | 0.413 | -0.1123 | No |
| 32 | IGFBP5 |  |  | 564 | 0.410 | -0.1147 | No |
| 33 | TNC |  |  | 613 | 0.393 | -0.1359 | No |
| 34 | FABP7 |  |  | 701 | 0.371 | -0.1765 | No |
| 35 | UBC |  |  | 792 | 0.348 | -0.2186 | No |
| 36 | METRN |  |  | 903 | 0.325 | -0.2708 | No |
| 37 | PDLIM4 |  |  | 982 | 0.313 | -0.3073 | No |
| 38 | SCRG1 |  |  | 1032 | 0.304 | -0.3295 | No |
| 39 | C4orf48 |  |  | 1209 | 0.278 | -0.4145 | No |
| 40 | HNRNPA2B1 |  |  | 1503 | -0.261 | -0.5572 | No |
| 41 | HSP90AA1 |  |  | 1535 | -0.268 | -0.5708 | No |
| 42 | MAD2L2 |  |  | 1539 | -0.269 | -0.5706 | No |
| 43 | MRPL51 |  |  | 1576 | -0.279 | -0.5866 | No |
| 44 | CDKN2C |  |  | 1593 | -0.285 | -0.5927 | No |
| 45 | RUVBL2 |  |  | 1599 | -0.286 | -0.5934 | No |
| 46 | HP1BP3 |  |  | 1618 | -0.290 | -0.6005 | No |
| 47 | FAM83D |  |  | 1624 | -0.292 | -0.6011 | No |
| 48 | HES6 |  |  | 1632 | -0.294 | -0.6027 | No |
| 49 | GAS2L3 |  |  | 1640 | -0.297 | -0.6043 | No |
| 50 | OIP5 |  |  | 1686 | -0.314 | -0.6246 | No |
| 51 | G2E3 |  |  | 1687 | -0.315 | -0.6226 | No |
| 52 | DCXR |  |  | 1689 | -0.315 | -0.6211 | No |
| 53 | NUDCD2 |  |  | 1691 | -0.316 | -0.6197 | No |
| 54 | TNFAIP8L1 |  |  | 1695 | -0.317 | -0.6192 | No |
| 55 | CNIH4 |  |  | 1751 | -0.340 | -0.6442 | No |
| 56 | RAB13 |  |  | 1761 | -0.345 | -0.6464 | No |
| 57 | RAN |  |  | 1772 | -0.350 | -0.6492 | No |
| 58 | HNRNPA3 |  |  | 1776 | -0.351 | -0.6485 | No |
| 59 | RBM8A |  |  | 1777 | -0.351 | -0.6463 | No |
| 60 | GLRX5 |  |  | 1778 | -0.351 | -0.6441 | No |
| 61 | KIF20A |  |  | 1792 | -0.358 | -0.6483 | No |
| 62 | POC1A |  |  | 1794 | -0.360 | -0.6466 | No |
| 63 | ILF2 |  |  | 1795 | -0.360 | -0.6443 | No |
| 64 | NEK2 |  |  | 1815 | -0.371 | -0.6514 | No |
| 65 | RHNO1 |  |  | 1824 | -0.377 | -0.6530 | No |
| 66 | C12orf75 |  |  | 1832 | -0.380 | -0.6541 | No |
| 67 | LDHA |  |  | 1833 | -0.380 | -0.6517 | No |
| 68 | EXOSC8 |  |  | 1865 | -0.397 | -0.6645 | No |
| 69 | TCP1 |  |  | 1875 | -0.401 | -0.6665 | Yes |
| 70 | CBX1 |  |  | 1878 | -0.405 | -0.6649 | Yes |
| 71 | RBMX |  |  | 1885 | -0.412 | -0.6653 | Yes |
| 72 | SHCBP1 |  |  | 1886 | -0.412 | -0.6628 | Yes |
| 73 | SAE1 |  |  | 1893 | -0.417 | -0.6631 | Yes |
| 74 | LIMA1 |  |  | 1899 | -0.421 | -0.6630 | Yes |
| 75 | MXD3 |  |  | 1905 | -0.428 | -0.6628 | Yes |
| 76 | SFPQ |  |  | 1908 | -0.429 | -0.6611 | Yes |
| 77 | HNRNPH3 |  |  | 1911 | -0.429 | -0.6594 | Yes |
| 78 | DCP2 |  |  | 1914 | -0.432 | -0.6577 | Yes |
| 79 | HMGA1 |  |  | 1915 | -0.433 | -0.6550 | Yes |
| 80 | EIF5 |  |  | 1923 | -0.439 | -0.6557 | Yes |
| 81 | DIAPH3 |  |  | 1924 | -0.439 | -0.6530 | Yes |
| 82 | STIP1 |  |  | 1933 | -0.446 | -0.6542 | Yes |
| 83 | MZT1 |  |  | 1936 | -0.447 | -0.6524 | Yes |
| 84 | SNRPB |  |  | 1937 | -0.447 | -0.6496 | Yes |
| 85 | HNRNPDL |  |  | 1940 | -0.448 | -0.6478 | Yes |
| 86 | CLIC1 |  |  | 1949 | -0.454 | -0.6489 | Yes |
| 87 | DDX39A |  |  | 1957 | -0.458 | -0.6495 | Yes |
| 88 | PTGES3 |  |  | 1970 | -0.467 | -0.6526 | Yes |
| 89 | TIMM10 |  |  | 1975 | -0.470 | -0.6516 | Yes |
| 90 | CMC2 |  |  | 1977 | -0.473 | -0.6492 | Yes |
| 91 | SPAG5 |  |  | 1997 | -0.490 | -0.6555 | Yes |
| 92 | PSMC3 |  |  | 1999 | -0.492 | -0.6529 | Yes |
| 93 | ARL6IP1 |  |  | 2001 | -0.494 | -0.6503 | Yes |
| 94 | TK1 |  |  | 2003 | -0.497 | -0.6477 | Yes |
| 95 | LRR1 |  |  | 2004 | -0.497 | -0.6446 | Yes |
| 96 | TUBB6 |  |  | 2006 | -0.500 | -0.6420 | Yes |
| 97 | CALM3 |  |  | 2022 | -0.516 | -0.6462 | Yes |
| 98 | RANBP1 |  |  | 2024 | -0.517 | -0.6435 | Yes |
| 99 | CACYBP |  |  | 2028 | -0.518 | -0.6417 | Yes |
| 100 | PARPBP |  |  | 2030 | -0.520 | -0.6390 | Yes |
| 101 | DEPDC1B |  |  | 2037 | -0.527 | -0.6387 | Yes |
| 102 | CDCA3 |  |  | 2041 | -0.529 | -0.6369 | Yes |
| 103 | CCT5 |  |  | 2042 | -0.529 | -0.6336 | Yes |
| 104 | TRIP13 |  |  | 2044 | -0.530 | -0.6308 | Yes |
| 105 | TROAP |  |  | 2046 | -0.533 | -0.6280 | Yes |
| 106 | TTK |  |  | 2047 | -0.533 | -0.6247 | Yes |
| 107 | KIF22 |  |  | 2053 | -0.541 | -0.6238 | Yes |
| 108 | STMN1 |  |  | 2063 | -0.567 | -0.6247 | Yes |
| 109 | SPDL1 |  |  | 2064 | -0.569 | -0.6211 | Yes |
| 110 | PTMA |  |  | 2065 | -0.570 | -0.6176 | Yes |
| 111 | ZWINT |  |  | 2069 | -0.577 | -0.6155 | Yes |
| 112 | RTKN2 |  |  | 2071 | -0.579 | -0.6124 | Yes |
| 113 | RACGAP1 |  |  | 2072 | -0.579 | -0.6088 | Yes |
| 114 | CENPA |  |  | 2073 | -0.586 | -0.6051 | Yes |
| 115 | LSM4 |  |  | 2076 | -0.587 | -0.6025 | Yes |
| 116 | SKA3 |  |  | 2077 | -0.590 | -0.5988 | Yes |
| 117 | BUB3 |  |  | 2078 | -0.596 | -0.5951 | Yes |
| 118 | AURKB |  |  | 2079 | -0.598 | -0.5914 | Yes |
| 119 | GGH |  |  | 2080 | -0.603 | -0.5876 | Yes |
| 120 | SRSF3 |  |  | 2081 | -0.606 | -0.5839 | Yes |
| 121 | CENPK |  |  | 2082 | -0.607 | -0.5801 | Yes |
| 122 | KNSTRN |  |  | 2085 | -0.615 | -0.5773 | Yes |
| 123 | CKAP5 |  |  | 2086 | -0.623 | -0.5734 | Yes |
| 124 | PLK1 |  |  | 2087 | -0.624 | -0.5695 | Yes |
| 125 | PIF1 |  |  | 2088 | -0.627 | -0.5656 | Yes |
| 126 | NCAPD2 |  |  | 2089 | -0.627 | -0.5617 | Yes |
| 127 | KIF18A |  |  | 2093 | -0.636 | -0.5592 | Yes |
| 128 | MIS18A |  |  | 2096 | -0.639 | -0.5563 | Yes |
| 129 | HMGN1 |  |  | 2099 | -0.646 | -0.5532 | Yes |
| 130 | PHF19 |  |  | 2101 | -0.650 | -0.5497 | Yes |
| 131 | SNRPD1 |  |  | 2105 | -0.653 | -0.5471 | Yes |
| 132 | CKAP2L |  |  | 2109 | -0.663 | -0.5445 | Yes |
| 133 | AURKA |  |  | 2110 | -0.673 | -0.5403 | Yes |
| 134 | RPA3 |  |  | 2112 | -0.678 | -0.5366 | Yes |
| 135 | LSM3 |  |  | 2114 | -0.694 | -0.5327 | Yes |
| 136 | CKS2 |  |  | 2115 | -0.694 | -0.5284 | Yes |
| 137 | CDC20 |  |  | 2117 | -0.695 | -0.5246 | Yes |
| 138 | RAD21 |  |  | 2118 | -0.697 | -0.5203 | Yes |
| 139 | ANP32B |  |  | 2119 | -0.700 | -0.5159 | Yes |
| 140 | CDCA2 |  |  | 2120 | -0.705 | -0.5115 | Yes |
| 141 | CENPM |  |  | 2125 | -0.709 | -0.5091 | Yes |
| 142 | CDCA8 |  |  | 2129 | -0.722 | -0.5061 | Yes |
| 143 | CKAP2 |  |  | 2131 | -0.729 | -0.5020 | Yes |
| 144 | KIF2C |  |  | 2136 | -0.756 | -0.4993 | Yes |
| 145 | KIF5B |  |  | 2137 | -0.756 | -0.4946 | Yes |
| 146 | KIF14 |  |  | 2141 | -0.773 | -0.4913 | Yes |
| 147 | NDC80 |  |  | 2142 | -0.775 | -0.4865 | Yes |
| 148 | H2AFV |  |  | 2143 | -0.779 | -0.4816 | Yes |
| 149 | FBXO5 |  |  | 2144 | -0.779 | -0.4768 | Yes |
| 150 | KIF23 |  |  | 2145 | -0.782 | -0.4719 | Yes |
| 151 | GMNN |  |  | 2146 | -0.785 | -0.4671 | Yes |
| 152 | MELK |  |  | 2148 | -0.786 | -0.4627 | Yes |
| 153 | ANLN |  |  | 2149 | -0.797 | -0.4577 | Yes |
| 154 | KIFC1 |  |  | 2151 | -0.802 | -0.4532 | Yes |
| 155 | DEPDC1 |  |  | 2152 | -0.809 | -0.4482 | Yes |
| 156 | DEK |  |  | 2154 | -0.817 | -0.4436 | Yes |
| 157 | TUBB |  |  | 2157 | -0.831 | -0.4394 | Yes |
| 158 | NUCKS1 |  |  | 2158 | -0.832 | -0.4343 | Yes |
| 159 | LSM5 |  |  | 2159 | -0.835 | -0.4291 | Yes |
| 160 | SERPINE1 |  |  | 2161 | -0.843 | -0.4243 | Yes |
| 161 | TUBB4B |  |  | 2162 | -0.843 | -0.4191 | Yes |
| 162 | ECT2 |  |  | 2163 | -0.846 | -0.4138 | Yes |
| 163 | HMGB3 |  |  | 2164 | -0.852 | -0.4085 | Yes |
| 164 | KIF20B |  |  | 2165 | -0.852 | -0.4032 | Yes |
| 165 | SPC24 |  |  | 2166 | -0.853 | -0.3979 | Yes |
| 166 | ARHGAP11A |  |  | 2167 | -0.855 | -0.3926 | Yes |
| 167 | DTYMK |  |  | 2168 | -0.858 | -0.3873 | Yes |
| 168 | BUB1 |  |  | 2170 | -0.860 | -0.3824 | Yes |
| 169 | FOXM1 |  |  | 2171 | -0.861 | -0.3771 | Yes |
| 170 | LBR |  |  | 2173 | -0.870 | -0.3722 | Yes |
| 171 | BUB1B |  |  | 2174 | -0.871 | -0.3668 | Yes |
| 172 | NUF2 |  |  | 2176 | -0.877 | -0.3618 | Yes |
| 173 | CEP55 |  |  | 2177 | -0.883 | -0.3563 | Yes |
| 174 | KPNA2 |  |  | 2178 | -0.894 | -0.3508 | Yes |
| 175 | HMMR |  |  | 2179 | -0.895 | -0.3452 | Yes |
| 176 | CCDC34 |  |  | 2180 | -0.898 | -0.3396 | Yes |
| 177 | CCNA2 |  |  | 2181 | -0.902 | -0.3340 | Yes |
| 178 | MIS18BP1 |  |  | 2183 | -0.903 | -0.3289 | Yes |
| 179 | KIF4A |  |  | 2186 | -0.921 | -0.3242 | Yes |
| 180 | PRR11 |  |  | 2187 | -0.930 | -0.3184 | Yes |
| 181 | CENPH |  |  | 2191 | -0.943 | -0.3140 | Yes |
| 182 | CENPW |  |  | 2192 | -0.943 | -0.3081 | Yes |
| 183 | TUBA1B |  |  | 2193 | -0.947 | -0.3023 | Yes |
| 184 | LMNB1 |  |  | 2194 | -0.950 | -0.2964 | Yes |
| 185 | HJURP |  |  | 2195 | -0.962 | -0.2904 | Yes |
| 186 | UBE2T |  |  | 2196 | -0.965 | -0.2844 | Yes |
| 187 | CDKN3 |  |  | 2199 | -0.979 | -0.2793 | Yes |
| 188 | CCNB2 |  |  | 2200 | -0.983 | -0.2732 | Yes |
| 189 | CKS1B |  |  | 2201 | -0.987 | -0.2670 | Yes |
| 190 | ESCO2 |  |  | 2204 | -1.004 | -0.2618 | Yes |
| 191 | SMC2 |  |  | 2205 | -1.006 | -0.2555 | Yes |
| 192 | KIF11 |  |  | 2206 | -1.008 | -0.2493 | Yes |
| 193 | SPC25 |  |  | 2208 | -1.019 | -0.2434 | Yes |
| 194 | CENPE |  |  | 2211 | -1.036 | -0.2380 | Yes |
| 195 | NUDT1 |  |  | 2214 | -1.054 | -0.2324 | Yes |
| 196 | CENPN |  |  | 2217 | -1.079 | -0.2267 | Yes |
| 197 | UBE2C |  |  | 2218 | -1.081 | -0.2200 | Yes |
| 198 | H2AFZ |  |  | 2220 | -1.090 | -0.2137 | Yes |
| 199 | HMGB1 |  |  | 2221 | -1.116 | -0.2067 | Yes |
| 200 | NCAPG |  |  | 2222 | -1.122 | -0.1998 | Yes |
| 201 | H2AFX |  |  | 2223 | -1.146 | -0.1926 | Yes |
| 202 | CCNB1 |  |  | 2224 | -1.148 | -0.1855 | Yes |
| 203 | CDK1 |  |  | 2225 | -1.153 | -0.1783 | Yes |
| 204 | PTTG1 |  |  | 2226 | -1.198 | -0.1709 | Yes |
| 205 | ANP32E |  |  | 2227 | -1.200 | -0.1634 | Yes |
| 206 | MAD2L1 |  |  | 2228 | -1.204 | -0.1560 | Yes |
| 207 | UBE2S |  |  | 2229 | -1.205 | -0.1485 | Yes |
| 208 | GTSE1 |  |  | 2230 | -1.220 | -0.1409 | Yes |
| 209 | DBF4 |  |  | 2232 | -1.247 | -0.1336 | Yes |
| 210 | HMGN2 |  |  | 2233 | -1.267 | -0.1257 | Yes |
| 211 | RRM2 |  |  | 2234 | -1.268 | -0.1179 | Yes |
| 212 | DLGAP5 |  |  | 2235 | -1.273 | -0.1100 | Yes |
| 213 | SMC4 |  |  | 2236 | -1.281 | -0.1020 | Yes |
| 214 | PBK |  |  | 2237 | -1.325 | -0.0938 | Yes |
| 215 | TMPO |  |  | 2239 | -1.382 | -0.0857 | Yes |
| 216 | PRC1 |  |  | 2240 | -1.397 | -0.0770 | Yes |
| 217 | HMGB2 |  |  | 2241 | -1.469 | -0.0678 | Yes |
| 218 | BIRC5 |  |  | 2242 | -1.469 | -0.0587 | Yes |
| 219 | ASPM |  |  | 2244 | -1.490 | -0.0499 | Yes |
| 220 | TOP2A |  |  | 2246 | -1.562 | -0.0407 | Yes |
| 221 | NUSAP1 |  |  | 2247 | -1.600 | -0.0308 | Yes |
| 222 | TPX2 |  |  | 2248 | -1.609 | -0.0208 | Yes |
| 223 | CENPF |  |  | 2249 | -1.691 | -0.0103 | Yes |
| 224 | MKI67 |  |  | 2252 | -1.892 | 0.0005 | Yes |
Table: GSEA details [plain text format]

  

Fig 2: FELDMAN\_2020\_G2-M: Random ES distribution      
 Gene set null distribution of ES for **FELDMAN\_2020\_G2-M**
